# Cercus Electric Stimulation Enables Cockroach with Trajectory Control and Spatial Cognition Training

**DOI:** 10.34133/cbsystems.0154

**Published:** 2025-03-07

**Authors:** Li Yu, Jieliang Zhao, Yufan Song, Zhiyun Ma, Zhong Liu, Lulu Liang, Mengdi Xu, Wenzhong Wang, Shaoze Yan

**Affiliations:** ^1^ School of Mechanical Engineering, Beijing Institute of Technology, Beijing 100081, P. R. China.; ^2^ School of Computer Science, Beijing Institute of Technology, Beijing 100081, P. R. China.; ^3^ School of Artificial Intelligence, Beijing Technology and Business University, Beijing 100048, P. R. China.; ^4^ Department of Mechanical Engineering, Carnegie Mellon University, Pittsburgh, PA 15213, USA.; ^5^ Department of Mechanical Engineering, Tsinghua University, Beijing 100084, P. R. China.

## Abstract

Cyborg insects are highly adaptable for detection and recognition assignments, achieved through the electrical stimulation of multiple organs and nerves to control their locomotion. However, it remains unclear whether these control strategies can promote memory formation in insects, thereby facilitating their training for recognition assignments. In this study, we employed a steering control strategy for cyborg insects in operant learning training of cockroaches in a T-maze. Remarkably, cockroaches developed a preference for specific maze channels after only five consecutive sessions of unilateral cercus electrical stimulation and steering behavior induction, achieving a memory score of 83.5%, outperforming traditional punishing training schemes. The experimental results confirmed the effectiveness of electrical stimulation on the cercus in improving the spatial cognition of cockroaches by inducing them to make specific choices in the maze. Our study revealed that the artificial locomotion control strategy can not only prompt insects to execute predetermined locomotion but also facilitate the formation of preferential memory for specific trajectories. Overall, our study highlights the electrical stimulation of sensory organs as a robust and efficient training protocol for spatial recognition learning in insects.

## Introduction

Cyborg insects have emerged as an ideal strategy and are anticipated to play a significant role in search and rescue operations in the future. They can efficiently navigate to designated locations by applying electrical stimulation to sensory organs, neurons, or muscular systems, achieving a high success rate in completing detection assignments that rely on portable sensors [1,2]. Indeed, insects exhibit odor detection [3–7], spatial discrimination [8,9], and visual recognition [10–14] capabilities based on their cognitive abilities, which eliminate the need for external sensing devices. These detection functions are achieved through the behavioral training of insects, which differs from the locomotion control strategy employed in cyborg insects. However, the learning efficiency of trained insects is inevitably influenced by individual differences and the design of the training scenarios. To date, no study has examined the efficacy of an electrical stimulation-based locomotion control strategy for the discrimination training of insects, such as spatial recognition in the maze.

By integrating learning features with rewards or publishing stimuli, insects can be trained in operant learning to select specific pathways within a maze based on their learning characteristics. The learning score—defined as the proportion of individuals approaching the correct channel—is adopted to evaluate training effectiveness. The selection of stimulus elements is based on the physiological characteristics of the trained insects. For cockroaches, typical punitive stimuli, including salt [15–17], electric shock [18], and strong light [19], have proven effective in operant learning within mazes and open arenas. Additionally, sucrose solution, a universal food reward, is widely applied to associate visual and odor cues to train selection behavior in the bifurcation maze of Hymenoptera insects and many other invertebrates [6,20–23]. Albeit serving as a quintessential reward stimulus, food-reward stimuli consistently exhibit substantial variations in learning scores among the different training individuals. This individual expression partially originates from differences in insect satiety [24]. Furthermore, genetic variation and aging contribute to individual differences in learning scores during operant learning with food, olfactory, visual, and mechanosensory stimuli [25–28]. Therefore, the impact of individual differences on the stability of insect learning performance remains an unresolved challenge.

In addition to individual differences, the shape of the bifurcation maze has a considerable impact on learning scores. In the operant learning of invertebrates, T-maze and Y-maze are commonly used as training scenarios. Both types of mazes incorporate a starting arm and two selection arms that are symmetrically distributed on both sides of the starting arm. The angle between the selection and starting arms in the Y-maze is 120°, while the selection arm offset is 90° from the starting arm in the T-maze. In the Y-maze, insects consistently demonstrate superior learning performance and produce higher memory scores than the T-maze [29], which may be because the bifurcation angle in the Y-maze matches the steering trajectory of insects in their natural state [30].

Electrical stimulation of the antenna and cercus is proficient in inducing insects to steer with an amplitude greater than that in the natural state. Unilateral cercus electrical stimulation initiates the steering escape response of insects by depolarizing giant fiber neurons that form synapses with wind-sensitive neurons of the cercus [31,32]. It has been confirmed that the steering locomotion parameters of cockroaches are intricately linked to electrical stimulation parameters [33,34]. Although the steering parameters induced by cercus stimulation are inevitably influenced by individual differences, the success rate of controlling cockroaches to turn and avoid obstacles remains consistently higher than 94% [35]. Recently, the steering control strategy of applying electrical stimulation to sensory organs has been widely utilized to guide cyborg insects to transverse complex mazes containing multiple T-shaped interactions [1,2,36–38]. However, the feasibility of electrical stimulation to sensory organs in training insects for spatial learning in mazes remains unclear.

To investigate the effect of electrical stimulation of sensory organs on insect memory formation, the cerci of cockroaches were stimulated with electric signals during training, inducing them to divert to a specific maze channel before making autonomous decisions. Before training, the gradient relationship between stimulus frequency and steering parameters was evaluated to obtain an electric pulse suitable for spatial learning. We demonstrated that cockroaches completed spatial learning after only five training sessions. The learning performance of cockroaches under electrical inducement-based training exceeded that of traditional punishment-based training. Our research demonstrated the effectiveness of electrical stimulation of sensory organs in operant spatial training of insects, which achieved ideal memory scores with minimal interference from individual differences.

## Materials and Methods

### Experimental animals

Adult Madagascar hissing cockroaches (*Gromphadorhina portentosa*) were used for operant conditioning and memory detection. Cockroaches were raised in sawdust-bedded plastic cases and fed with carrots and oatmeal. The breeding chambers were maintained at an ambient temperature of 25 °C and a relative humidity of 60%.

### Construction of training apparatus

**Fig. 1.**
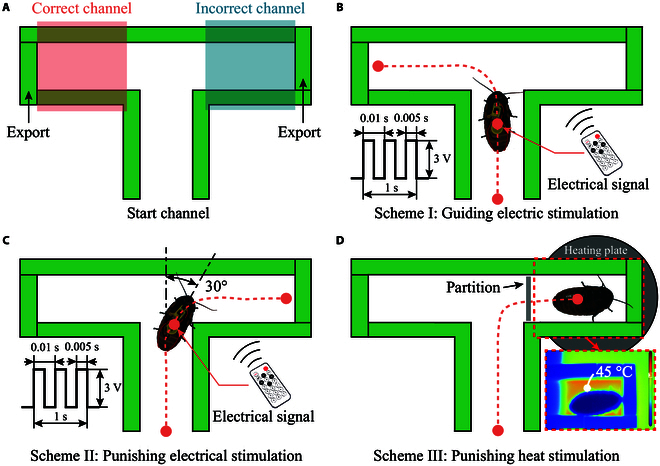
Maze channel design and implementation methods for the three training schemes. (A) Maze channel design, (B) guiding electric stimulation, (C) punishing electric stimulation, and (D) punishing heat stimulation. The lower-right image is a thermal image of the maze surface. The pink dotted lines indicate the movement trajectories of the cockroach in the maze.

**Fig. 2.**
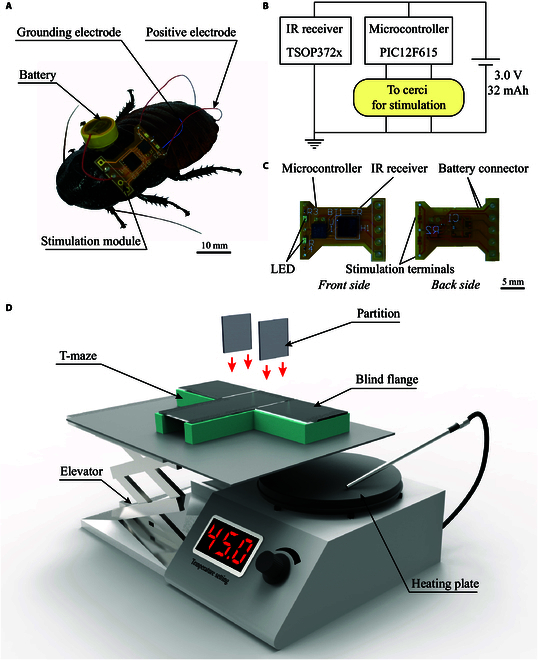
Construction of electrical and heat conditioning. (A) The cyborg insect used for electrical stimulation training consisted of a living Madagascar hissing cockroach and a wireless stimulation module. (B) Circuit diagram of the wireless stimulation module. (C) Composition of the wireless stimulation module. (D) Training apparatus used for heat-stimulation training.

The cockroaches were trained to select a particular target channel in a custom-made T-maze. A maze with two target channels and a start channel (inner dimensions: 92 mm long, 40 mm wide, and 20 mm high for the target channels, and 80 mm long, 40 mm wide, and 20 mm high for the start channel) was built using green foam building blocks (Fig. 1A). Rectangular blocks serving as exports at the end of the two target channels could be removed when the cockroach completed the target channel selection. The maze was immobilized on a gray acrylic board (3 mm thickness), and the surface of each channel was covered with transparent acrylic boards (Fig. 2D). The inner width was designed to fit the average width of cockroaches to avoid walking out of the maze, thereby increasing the training success rate.

### Training protocols and retention tests

In the operant training of cockroaches, we used the electrical pulse signal applied to the cercus as a penalty element. Behavioral training involving electrical stimulation comprised two schemes: guiding and punishing electrical stimulation. In the former, electrical stimulation was applied to the unilateral cercus before the cockroach made an autonomous choice at the fork in the maze, inducing it to turn to a specific target channel (Scheme I and Fig. 1B). The latter was characterized by the same electrical stimulation on the cercus applied after the sample made a choice, serving as punitive feedback for incorrect selection (Scheme II and Fig. 1C). The incorrect selection was manifested when the angle between the body axis of the cockroach and the center of the maze was greater than 30° (Fig. 1C). To compare the learning effects of electrical stimulation, we trained cockroach selection behavior in a maze using heat punishment (Scheme III). In this scheme, individuals were punished for selecting an incorrect channel (Fig. 1D). Before training, we verified whether cockroaches exhibited a natural preference for maze-specific channels under no-intervention conditions.

During operant conditioning training, the experimental individuals were first placed at the entrance of the start channel of the maze. The cockroaches were gently pushed if they remained stationary for > 5 min. Subsequently, the cockroach was gently pushed out of the channel after it accomplished selection and entered the target channels. Each training scenario and control experiment involved the participation of 10 active adult individuals with body lengths of 5–7 cm. Regardless of the training method, each training protocol consisted of five trials with a time interval of 30 min. Short-term retention tests (sRTs) and long-term retention tests (lRTs) were conducted for 30 min and 24 h after the five training trials, respectively. In the retention test, cockroaches were allowed to make five consecutive autonomous choices in the T-maze without external stimuli or interventions.

### Achievement of thermal punishment and electrical punishment

The heating penalty experimental system included a T-maze, elevator, partitions, blind flanges, and heating plate (Fig. 2D). In each training session, if the cockroach selected the incorrect target channel, the channel was closed by the partition, and the bottom of the maze channel dropped down to the surface of the heating plate through the elevator. The temperature of the heating plate was set to 45 °C, which is higher than the suitable survival temperature for cockroaches (15–37 °C). The experimental individuals remained in a closed heating channel for 5 min. Consequently, the export point of the incorrect channel was opened, and the cockroach was gently expelled.

Electrical punishment was accomplished by constructing a wireless stimulation module and a cyborg cockroach (Fig. 2A). To stimulate the swerving locomotion of cockroaches, a wireless microstimulator was assembled to receive wireless commands and generate electrical pulses. The microstimulator consisted of a base circuit, lithium-manganese battery (cr927, 3 V, 600 mg, 32 mAh, XINLGO), infrared (IR) receiver (TSOP372X, 35 mg, VISHAY), microcontroller (PIC12F615, 23 mg, Microchip), two light-emitting diodes (19-217/R6C-X, 0603 package size, EVERLIGHT), capacitor, and three resistors (0201 package size). The circuit diagram and the appearance of the stimulation module are illustrated in Fig. 2B and C. The circuit board of the microstimulator was processed by double-sided screen printing on a PET (polyethylene terephthalate) substrate. The battery and assembled stimulation module were immobilized on the thorax of the cockroach using foam double-sided adhesive tape (Fig. 2A). The total weight of the finished stimulating backpack was approximately 675 mg, which was much lower than the maximum load capacity of cockroaches. Consequently, we inferred that the stimulation backpack did not interfere with cockroach locomotion. Teflon-coated silver wires (bare, 100 μm diameter; coated, 250 μm diameter) were implanted as stimulating electrodes. Two positive electrodes and one grounding electrode were inserted into two cerci and the fourth abdominal segment, respectively (Fig. 2A). The implantation depths of the positive and grounding electrodes were 10 and 5 mm, respectively. The detailed assembly process of the cyborg cockroach is presented in Movie S1.

### Determination of electrical stimulation parameters for spatial learning

The cockroach exhibits a steering response under electrical stimulation on the unilateral cercus, whose steering locomotion parameters are associated with the electrical stimulation parameters applied to cyborg insects. It has been confirmed that pulse frequency significantly affects the steering parameters of both crawling and flying insects, including angular displacement [39,40], radius [41], angular velocity [42], lateral acceleration [43], and lateral force [41]. This study specifically focused on the influence of frequency on the turning radius and angular displacement. We aimed to obtain the optimal parameter with the maximum angular displacement and relatively small radius for electrical stimulation-based training and ensure that the cockroach undergoing the guiding electric stimulation training scheme can successfully turn to the correct channel during operant learning. Except for the frequency, the amplitude, burst width, and pulse duration used for the electrical inducement were set to 3 V, 50%, and 1 s, respectively. To eliminate the impact of the stimulus parameters on training efficacy, the signal used in the guiding electric stimulation training scheme was identical to that used in the punishing electric stimulation training scheme. Six cockroaches were randomly exposed to 10 frequencies ranging from 10 to 100 Hz, with an interval of 10 Hz. Pulse-wave signals applied to the unilateral cercus were generated using a pulse stimulator (MODEL 2100, A-M SYSTEMS). The response behavior of the individuals was recorded using a digital single-lens reflex (DSLR) camera (D850, Nikon, Japan) at 60 frames per second (fps). The recovery period between the two stimulations was 5 min.

## Results

### The steering response under electrical stimulation of unilateral cercus

**Fig. 3.**
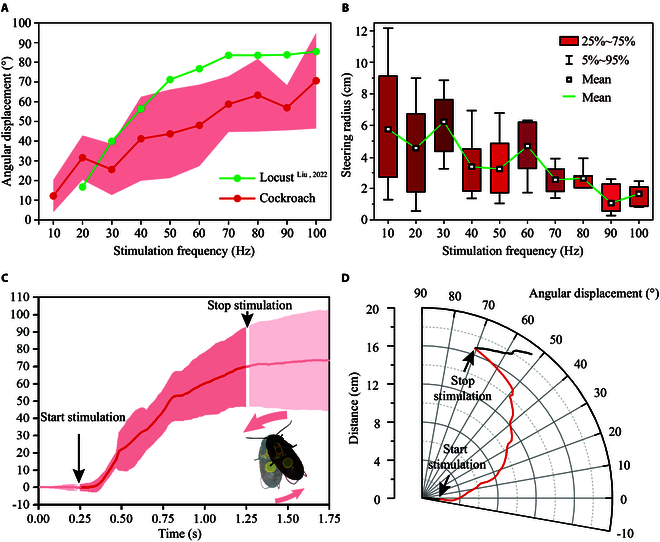
Steering response of cockroaches to electrical stimulation of the unilateral cercus. (A) The angular displacement of cockroaches when the frequency of stimulation on the cercus varied from 10 to 100 Hz (*N* = 6 cockroaches, *n* = 114 trials), and angular displacement of locusts when the frequency of stimulation on antennae varied from 20 to 100 Hz [44]. (B) Steering radius of cockroaches when the stimulation on the cercus varied from 10 to 100 Hz (*N* = 6 cockroaches, *n* = 114 trials). (C and D) Steering response of cockroaches to unilateral cercus stimulation at 100 Hz [(C), *N* = 6 cockroaches, *n* = 11 trials]. The shaded regions in (A) and (C) represent ±1 standard deviation.

Stimulation applied to the unilateral cercus induced the cockroach to flex its tail to the opposite side of the stimulus, thus aligning the body axis with the stimulus. The cockroach rotated 70.63° ± 24.17° counterclockwise when stimulated with 100 Hz on the left cercus (Fig. 3C, *N* = 6 cockroaches, *n* = 11 trials). The cockroach did not perform obvious sideways crawling during the unilateral stimulus (Fig. 3D). The variation in the body direction became smooth immediately after stimulation termination (Fig. 3C).

The angular displacement and steering radius were correlated with the electrical pulse frequency (Figs. 3A, B, and S1). As the pulse frequency increased, the angular displacement exhibited an upward trend (Pearson's correction test, *ρ* = 0.957, *P* < 0.01) (Movie S2). Conversely, the steering radius exhibited a downward trend (Pearson’s correction test, *ρ* = −0.865, *P* < 0.01), which was more significant in the 60–100 Hz interval. Notably, the average steering angle at frequencies of 30 and 90 Hz did not completely align with the increasing trend (Fig. 3A), which may be attributed to slight sample differences at each frequency and the attenuation of cockroach physiological activity due to multiple stimuli. These factors also explain the fluctuation in the steering radius in the range of 10–60 Hz (Fig. 3B) and significant standard deviations of the steering parameters. The same standard deviation scale of locomotion parameters was also presented by Liu et al. [44], who studied the omnidirectional jump control of cyborg locusts.

Steering locomotion under the cercus electrical stimulus results in the activation of wind-sensitive sensory neurons, which cover the surface of the cerci and are responsible for initiating escape behavior toward approaching predators [45]. The graded response of steering escape may be attributed to a correlation between the firing rate of sensory interneurons and the frequency of applied stimulation [33,46], thereby modifying the willingness to escape behavior. In addition to cockroaches, the dependence of angular displacement on the stimulation frequency has been observed in various insects, such as locusts (Fig. 3A).

The graded steering response for different frequencies provides the potential for precisely regulating cockroach behavior and a reference for selecting the appropriate parameters for electrical inducement-based operant conditioning. The structural and dimensional characteristics of the T-maze revealed that the electrical pulse that can induce steering locomotion with greater angular amplitude and relatively small radius (smaller than the inner width of the maze) can increase the probability of cockroaches steering to the correct channel in operant training. Therefore, the frequency of 100 Hz with a maximum average angular amplitude of 70.63° ± 24.17° and an average radius of 1.62 ± 0.69 cm was selected to participate in the electrical stimulation-based operant conditioning (Fig. 3A and B).

### The learning performance of cockroaches based on guiding electric stimulation training

**Fig. 4.**
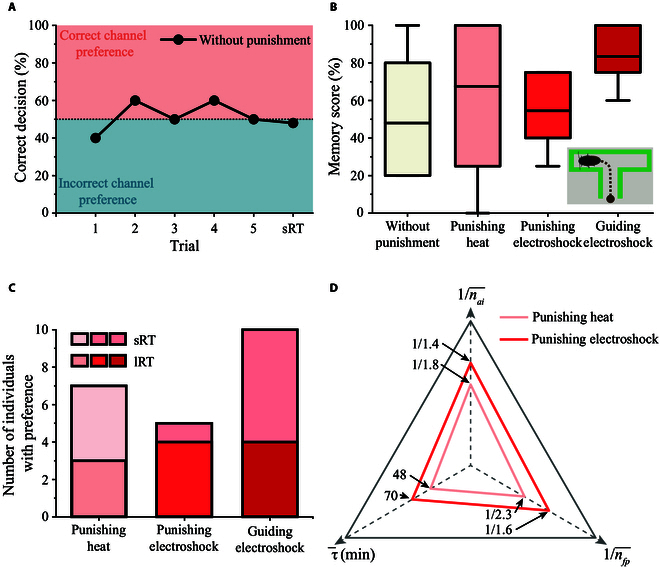
The learning performance of cockroaches in operant conditioning training. (A) Selection behavior of individuals without punishment. (B) Responsive scores of individuals in the control experiments and training. (C) Number of individuals with preferences for different training schemes. The borders of the boxes represent the 25th and 75th percentiles. The average value is presented as a horizontal line. The error bar represents the 1.5 interquartile range. (D) $\bar{n_{\mathrm{ai}}}$ (average number of stimuli for the cockroach to avoid the incorrect channel), $\bar{n_{\mathrm{fp}}}$ (average number of stimuli for individuals to form preference), and $\bar{\tau }$ (average duration of punishment memory) of training under punishing heat and punishing electroshock.

Electrical stimulation of the unilateral cercus has been demonstrated to be effective for steering control of cockroaches. When a left-steering stimulus was applied to the cockroach located at the end of the starting channel in the maze, the cockroach promptly steered toward the left channel (Fig. 1B and Movie S4). Based on this experimental phenomenon, we first investigated whether a cockroach would develop a preferential memory for a specific channel in the maze if it was artificially induced to enter a specific channel. Therefore, a left-turn electrical stimulus was applied to the cockroach during each training session before making decisions at the end of the starting channel in the T-maze (Fig. 1B). After the five training trials, we assessed the formation of short-term memory by calculating the memory score, defined as the average proportion of 10 individuals choosing the left-correct channel in the memory test. In the short-term memory detection test, cockroaches that received successive left-turn electric inductions demonstrated a significant preference for the left channel compared to their unstimulated counterparts. The average memory score reached 85.50% ± 0.15% (Fig. 4A and B). Furthermore, we quantified the number of samples with a preference for the left channel in both sRT and lRT. All samples from the 10 individuals receiving guiding electric stimulation training exhibited a preference for the left channel 30 min after training, whereas only four samples maintained the same preference 24 h after training (Fig. 4C).

### The learning performance of cockroaches based on punishing electric stimulation training

We investigated the electrical stimulation applied to the cercus as penalty feedback only when the cockroaches made inaccurate autonomous choices in operant spatial training. During the training process, an electrical pulse was applied to the cercus when the cockroach had a clear tendency to turn to the incorrect right channel (Fig. 1C). Conversely, the cockroach was not stimulated when the correct left channel was selected. Unlike the cockroach that received guiding electrical stimulation, the cockroach that received stimulation after choosing the intersection did not change its locomotion direction because the maze size limited the steering behavior of the cockroach (Fig. 1C and Movie S3).

To compare the training efficacy of the punishing electroshock with conventional punishing stimulation modalities, an additional 10 samples were subjected to heat exposure when steered toward the right-incorrect channel during training (Fig. 1D). Throughout the operant learning, the cockroach avoided an incorrect channel with punishment after receiving one or more stimuli. We defined the parameters *n*_*ai*_ (the number of stimuli for the cockroach to avoid the incorrect channel), *n*_*fp*_ (the number of stimuli for the cockroach to form a preference for the correct channel in sRT), and *τ* (maximum memory duration for the penalty) to describe the operant learning performance of cockroaches in the two training modalities. Notably, *τ* was applicable only to the cockroach that made an incorrect choice in the first training trial because the calculated memory duration might be less than the actual value for the cockroach that was selected accurately for the first time. Learning performance in both training modalities and memory scores in the retention tests are presented in Table 1.

**Table 1.** The learning performance and memory scores of cockroaches under electric shock and heat punishment.

Training modalities	Individual	Selection in each training session					Memory score in sRT (%)	Memory score in lRT (%)	*τ* (min)
		1st	2nd	3rd	4th	5th			
Punishing electric stimulation	1	√ ^a^	**×** ^b^	√	√	√	75	25	* ^c^
	2	√	√	√	**×**	√	75	75	*
	3	√	√	√	**×**	√	40	*	*
	4	**×**	√	√	√	×	75	100	90
	5	√	**×**	**×**	√	√	60	100	*
	6	√	**×**	√	**×**	√	75	100	*
	7	×	√	√	×	×	40	*	60
	8	**×**	√	√	**×**	**×**	40	*	60
	9	√	**×**	**×**	**×**	√	40	*	*
	10	√	**×**	**×**	√	**×**	25	*	*
Punishing heat stimulation	I	√	√	**×**	√	√	100	100	*
	II	√	√	√	√	**×**	100	40	*
	III	√	**×**	**×**	√	√	100	40	*
	IV	√	√	×	×	√	75	75	*
	V	**×**	**×**	**×**	√	√	0	0	60
	VI	**×**	√	**×**	√	**×**	25	0	30
	VII	**×**	√	√	**×**	**×**	100	100	60
	VIII	**×**	√	√	**×**	**×**	25	*	60
	IX	√	**×**	**×**	**×**	√	75	0	*
	X	×	×	×	√	×	75	40	30

^a^ √ indicates that the individual selected the correct channel.

^b^ × indicates that the individual selected the incorrect channel and received punishment.

^c^ * indicates that these data are unavailable for this individual.

Statistical results indicated that the average number of penalty sessions required for the cockroach to begin avoiding the electric-associated channel was 1.4, which was lower than that required for the cockroach to begin avoiding the thermal-associated channel (Fig. 4D). Compared with thermal stimulation, cockroaches exhibited a longer memory duration for punishing electric stimulation. However, the electrical stimulus was applied much shorter than the heat stimulus. Consequently, the average number of electrical stimuli for cockroaches with a short-term preference for the correct channel was less than that for heat stimuli. However, the average memory score obtained through punishing electroshock training was slightly lower than that obtained through punishing heat training, only 54% (Fig. 4A). This indicates that the samples slightly preferred the correct channel. The number of individuals developing a short-term preference for the left-correct channel through punishing electroshock training was also lower than that through thermal penalty training (Fig. 4C). However, in the lRT, four samples trained with punishing electrical stimulation retained preferential memory, whereas only three of eight samples subjected to heat training that exhibited short-term preferential memory remained in preference for the left-correct channel.

## Discussion

### Guiding electric stimulation training performs well in the operant learning of cockroaches for T-maze

The present study describes the learning performance of cockroaches in the T-maze based on three different training modalities. Among these three training approaches, the training protocol based on guiding electroshock obtained the highest memory score of 83.50% ± 0.15%, significantly higher than that of punishing electroshock and punishing heat training. Besides, all cockroaches participating in the guiding electroshock training exhibited a preference for the left-correct channel in the sRT. Regarding the memory score and sample size with correct preference, guiding electroshock training was more effective than punishing stimulation-based training. In the guiding electroshock training, the choice of cockroach in each session was influenced by the electrical signal acting on the left cercus, whereas in the punishing stimulation-based training, the number of stimuli the cockroach received varied according to its choice. Therefore, we hypothesized that the memory score of punishing-stimulation-based training could be enhanced by associating a positive feedback stimulus with the correct channel. The guiding electroshock training protocol we established effectively avoided training failure due to a decrease in the insects’ desire to explore the maze compared to the traditional punishing and rewarding-based training protocol.

Electroshock has recently become a common modality as a negative reinforcer for operant learning of insects in mazes [47–49]. Previously, electrical penalties were typically administered using a power source fixed on the surface of training scenarios. A power source was used to deliver shocks to the foot pads of the insects when they made error selections [50]. This strategy was incorporated by Barraco et al. to train the selection behavior of cockroaches in the T-maze, resulting in a criterion of 83% [18]. Although the memory score was comparable to that obtained using guiding electroshock training, the cockroach underwent training sessions that included up to 20 trials, which was significantly higher than the training sessions in our modality. Moreover, electrical stimulation of the unilateral cercus activated avoidance behavior toward approaching predators, which is distinguished from the rather unnatural electrical stimulus applied to the footpads of insects.

### The learning performance obtained through cercus electrical stimulation training possesses a stronger stability

To compare the stability and robustness of the training modalities adopted for memory formation in insects, we calculated the variance of the sample memory scores for the different training protocols (Fig. 4B). The calculation results demonstrated that the dispersion in memory scores of the two electrical stimulation-based trainings was significantly less than that of thermal stimulation-based training. The variance in the memory score for guiding electroshock training was only 0.022. Additionally, 10 individuals participating in guiding electroshock training acquired a short-term preference for a specific channel of the maze, exceeding the number of samples that obtained preferential memory under punishing electric stimulation and punishing heat stimulation training (Fig. 4B). Therefore, the statistical results demonstrated a higher stability of learning performance obtained by guiding electroshock in the sample population involved in the training. However, the variance in learning performance caused by individual differences in cockroaches could not be completely resolved owing to the random selection of samples in the training experiments. It has been demonstrated that genetic variation [25], neuronal plasticity difference [51–53], and aging [27] all contribute to the difference in learning speed and memory ability of insects, further triggering bias in learning performance. Therefore, it is challenging to eliminate learning performance fluctuations caused by individual differences using a single fixed training mode. In the future, we will adjust the intensity of electrical stimulation and the frequency and number of applied electroshocks to accommodate the differences in the learning speed of different individuals, thereby further reducing the individual variability in memory scores.

## Conclusion

This study demonstrated the efficacy of the cyborg insect locomotion control technique for spatial recognition training in maze navigation in cockroaches. Specifically, the development of preferential memory in the bifurcation maze was facilitated by electrical stimulation of the unilateral cercus, which prompted cockroaches to navigate toward a specific channel within the maze. The guiding electric stimulation training strategy achieved an exceptional memory score of 83.50% with only five training sessions, significantly less than the traditional punishing operant training protocol [18,54].

Using the innate neuroplasticity and learning capabilities of insects, recognition and selection training enables cyborg insects to swiftly and accurately identify target objects in real search scenarios. Moreover, it enables insects to adjust and optimize search paths, thereby improving the efficiency of search and rescue operations. Our research highlights the impact of continuous cercus electroshock, a cyborg insect locomotion control strategy, on cockroach memory formation. It also provides innovative insights into behavioral training modalities for insects.

In future studies, we aim to integrate sensory cues with cercus electroshock further to enhance cyborg insects’ ability to recognize environmental, visual, and odor information. This will expand the applications of cyborg insects for detection assignments.

## Data Availability

Data supporting the findings of this study are available in the main text or the Supplementary Materials.
