# Supplementary material for: Cercus Electric Stimulation Enables Cockroach with Trajectory Control and Spatial Cognition Training
Source: Cyborg Bionic Syst. 2025 Mar 7;6:0154. doi: 10.34133/cbsystems.0154 (PMC11886356; doi:10.34133/cbsystems.0154)
Supplement: Supplementary 1 — Fig. S1 Movies S1 to S4 [file cbsystems.0154.f1.zip › Supplementary Material.docx]

Supplementary Materials

The model equations between locomotion parameters and frequency

The relationship between the effect of frequency (*f*) on the movement parameters (angular displacement, *θ*; steering radius, *r*) and the extent of the effect can be described by linear regression (Figure S1). The model equations between angular displacement and frequency, and steering radius and frequency are, respectively:, (R=0.915); ,(R=0.748). The model equations demostrated that

frequency had a significant positive and negative relationship on angular displacement and turning radius, respectively. In addition, these models can be used to predict the motion parameters of cockroaches at different frequencies, providing possibilities for realizing the precise regulation of cockroach motion. However, the accuracy of the models is affected by the current experimental sample size, and the predictive effects of the models on the regulation of insect behavior are also affected by the individual differences of insects.


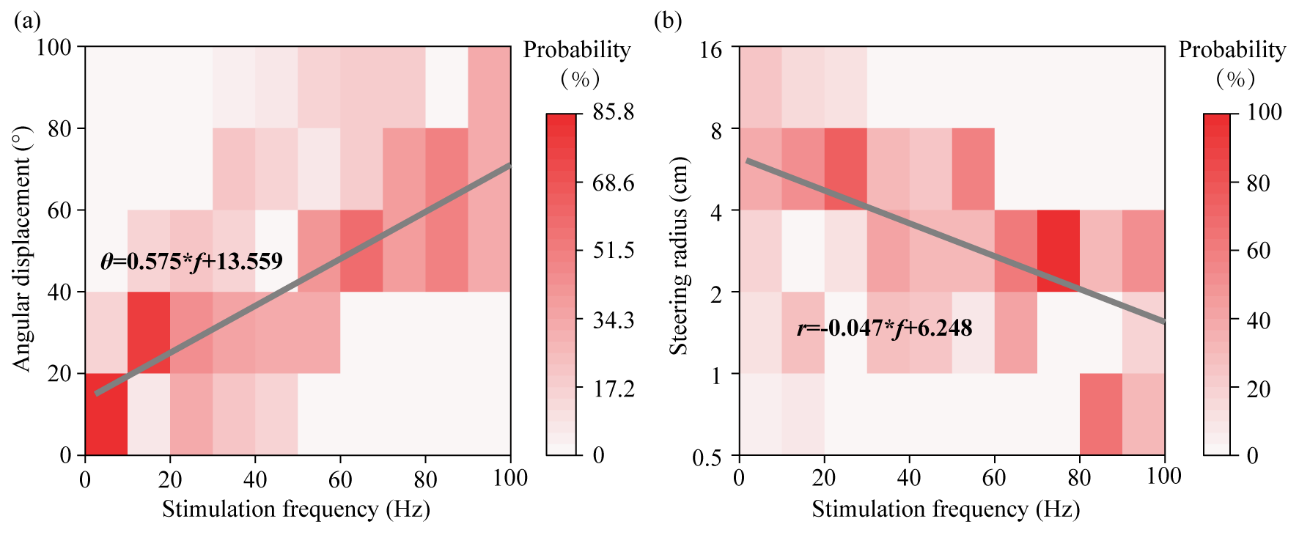


**Figure S1. Distribution of angular displacement (*θ*) and steering radius (*r*) at different stimulation frequencies (*f*).** The black straight lines in (a)/ (b) fit the linear correlation between the angular displacement/steering radius and the stimulus frequency, respectively.

Figures S1 #

Movies S1 to S4#
